# Post-Cardiotomy Blood Parameters as a Prognostic Marker in Acute Aortic Dissection Surgery

**DOI:** 10.21470/1678-9741-2024-0087

**Published:** 2025-03-13

**Authors:** Mesut Engin, Umut Serhat Sanrı, Ufuk Aydın, Yusuf Ata, Senol Yavuz

**Affiliations:** 1 Department of Cardiovascular Surgery, University of Health Sciences, Bursa Yuksek Ihtisas Training and Research Hospital, Yildirim, Bursa, Turkey. E-mail: mesut_kvc_cor@hotmail.com; 2 Department of Cardiovascular Surgery, University of Health Sciences, Bursa Yuksek Ihtisas Training and Research Hospital, Yildirim, Bursa, Turkey; 3 Department of Cardiovascular Surgery, University of Health Sciences, Bursa Yuksek Ihtisas Training and Research Hospital, Yildirim, Bursa, Turkey; 4 Department of Cardiovascular Surgery, University of Health Sciences, Bursa Yuksek Ihtisas Training and Research Hospital, Yildirim, Bursa, Turkey; 5 Department of Cardiovascular Surgery, University of Health Sciences, Bursa Yuksek Ihtisas Training and Research Hospital, Yildirim, Bursa, Turkey

Dear Editor,

We have read the article by Hu et al.^[1]^ entitled “Postoperative Prognostic Nutritional Index and Fibrinogen Could Well Predict Poor Prognosis of Acute Type A Aortic Dissection Patients After Surgery” with great interest. First of all, we congratulate the authors on their good contribution to the literature. However, we would like to discuss some points about acute type A aortic dissection surgery and postoperative complications.

Various markers obtained from routine blood values are being investigated in the cardiovascular field as well as in many fields of medicine. Prognostic studies can be conducted by evaluating these values preoperatively and at various postoperative times. However, in surgeries performed with cardiopulmonary bypass (CPB), blood values are affected by extracorporeal circulation^[2]^. Fibrinogen values may also vary depending on the time elapsed after leaving CPB^[3]^. Therefore, it is important at what time after CPB blood evaluations are made. In the current study, the authors investigated the prognostic importance of prognostic nutritional index (PNI) and fibrinogen values in the first 24 hours postoperatively^[1]^. However, it should not be forgotten that the evaluations made at the sixth hour and those made at the 24^th^ hour may contain differences. It may be misleading that this period contains significant differences across the entire patient group. Albumin is an important negative acute phase reactant used in PNI calculation. In some clinics, albumin can be used in CPB systems. Thus, clinical results have been reported that fluid requirement and pulmonary edema in patients are reduced^[4]^. Did the authors use albumin in the preparation of prime solution in all patients within the specified dates? Or was albumin given to patients within the first 24 hours? Similarly, it is also important whether patients were given fibrinogen supplements in the preoperative period. In a study conducted by Li et al.^[5]^, fibrinogen supplementation in the preoperative period was shown to reduce postoperative complications.

The presence of malperfusion is an important condition that affects postoperative results in aortic dissection patients. Malperfusion can occur in many forms including cerebral malperfusion, peripheral malperfusion, visceral malperfusion, involvement of supra-aortic branches, coronary malperfusion, and renal malperfusion^[6]^. In the current study, the presence of malperfusion was evaluated under a single heading and was found to be associated with prolonged ventilation^[1]^. However, it may be misleading to expect that cerebral or visceral organ malperfusion and lower extremity peripheral artery malperfusion may produce similar clinical results.
